# Identification of Novel B Cell Epitopes on the Nucleocapsid Protein of Porcine Epidemic Diarrhea Virus

**DOI:** 10.3390/v18030309

**Published:** 2026-03-02

**Authors:** Ruiying Wang, Meng Zhong, Ye Liu, Zichen Gao, Jianing Hu, Haiyan Zhang, Qingtao Liu, Bin Zhou, Xiuli Feng

**Affiliations:** 1 Key Laboratory of Animal Microbiology of China’s Ministry of Agriculture, College of Veterinary Medicine, Nanjing Agricultural University, Nanjing 210095, China; 2023107109@stu.njau.edu.cn (R.W.); 2023807130@stu.njau.edu.cn (M.Z.); 2023007051@stu.njau.edu.cn (Y.L.); 2022107058@njau.edu.cn (Z.G.); 2021207047@stu.njau.edu.cn (J.H.); 2024107041@stu.njau.edu.cn (H.Z.); zhoubin@njau.edu.cn (B.Z.); 2 MOE Joint International Research Laboratory of Animal Health and Food Safety, College of Veterinary Medicine, Nanjing Agricultural University, Nanjing 210095, China; 3 Key Laboratory of Veterinary Biological Engineering and Technology, Ministry of Agriculture, Institute of Veterinary Medicine, Jiangsu Academy of Agricultural Sciences, Nanjing 210014, China; taoqingliu2013@163.com

**Keywords:** porcine epidemic diarrhea virus (PEDV), nucleocapsid protein, monoclonal antibody, epitope mapping, B-cell epitope

## Abstract

Porcine epidemic diarrhea (PED), caused by the porcine epidemic diarrhea virus (PEDV), is an acute and highly contagious intestinal disease that inflicts substantial economic losses on the global swine industry. The nucleocapsid (N) protein of PEDV plays a critical role during viral infection and replication. In this study, the full-length *N gene* was cloned and expressed using the prokaryotic expression vector pET-32a (+). The purified recombinant N protein was used to immunize BALB/c mice. Subsequently, splenocytes from the immunized mice were fused with SP2/0 cells, and hybridoma cell lines secreting monoclonal antibodies (mAbs) against N protein were screened via indirect ELISA. The linear B-cell epitopes recognized by the mAbs were mapped using truncated N protein fragments. Results showed that three stable hybridoma cell lines (1A3, 1G1 and 1A10) secreting N protein-specific mAbs were obtained. Epitope mapping revealed that mAbs 1A3 and 1G1 recognized the epitope ^71^SNWHF^75^, whereas mAb 1A10 recognized ^66^RIEQP^70^. Bioinformatics analysis indicated that these epitopes are highly conserved among the analyzed PEDV strains and show no cross-reactivity with the N proteins of other coronaviruses. These findings could provide valuable experimental materials for further investigation of the N protein’s structure and function and support the development of diagnostic assays and subunit antigen vaccine for PEDV.

## 1. Introduction

The Porcine epidemic diarrhea (PED) was first reported in the United Kingdom (1977) and Belgium (1978), and gradually became endemic in numerous countries throughout Asia and the Americas [1,2]. The disease was first identified in China in 1980. PEDV infections were generally sporadic and limited in scope. However, in October 2010, a large-scale PED outbreak emerged, caused by a highly pathogenic GII variant strain in China, which severely impacted the swine industry and resulted in substantial economic losses [3]. Based on the homology of the hypervariable S1 region within N-terminal portion of the spike (S) gene, PEDV is primarily classified into two major genotypes, GI and GII. The GI genotype is further subdivided into GIa and GIb, while GII comprises GIIa and GIIb. Before 2010, the less virulent GI genotype was predominant; however, GII strains currently represent the majority of circulating isolates in China [4,5,6,7,8].

PEDV is an enveloped, single-stranded, positive-sense RNA virus with a genome approximately 28 kb. The viral genome consists of 5’ and 3’ untranslated regions (UTRs) and seven open reading frames (ORFs), four of which encode the major structural proteins: the spike (S), envelope (E), membrane (M), and nucleocapsid (N) [9]. The N protein is composed of 441 amino acids and exhibits high sequence conservation among viral strains, consisting of two domains: the N-terminal domain (NTD) and the C-terminal domain (CTD) [10]. Among the structural proteins, N protein is the most abundant both in the virion and within infected cells. Antibodies against N protein are generated early following PEDV infection in pigs, making it a suitable target protein for early infection detection and diagnosis applications [11].

N protein fulfills multiple functions during viral infection, including non-specifically binding to viral RNA during replication and forming the nucleocapsid complex. N protein also facilitates viral transcription by interacting with translation initiation factors and promoting the circularization of viral mRNA [12]. Furthermore, N protein induces S-phase arrest in host cells that creates a favorable environment for viral replication [13,14], and antagonizes type I interferon (IFN) production, thereby contributing to viral evasion [15,16]. Additionally, N protein has also been shown to promote the ubiquitination of both viral components and host antiviral proteins, modulating virus–host interactions and innate immune responses [17,18].

Current challenges in PEDV control include insufficient cross-protection afforded by existing vaccines and limited diagnostic specificity [19]. The identification of novel B-cell epitopes within N protein is crucial for developing improved PEDV diagnostics and vaccines, and also provides valuable tools for investigating viral infection mechanisms. In this study, the N protein of PEDV was expressed using a prokaryotic system. BALB/c mice were immunized with the purified recombinant N protein, and the splenocytes from immunized mice were fused with SP2/0 cells to generate monoclonal antibodies (mAbs) targeting N protein. Linear epitopes within the N protein were identified via Western blot analysis, leading to the characterization of three epitopes, including two novel ones. Homology and antigenicity analyses of these epitopes provide valuable resources for further elucidating the functions of the N protein, and support future vaccine design and diagnostic development.

## 2. Materials and Methods

### 2.1. Virus and Cells

The PEDV isolate was kindly provided by Professor Zhou Bin from Nanjing Agricultural University, which was stored at −80 °C in our laboratory.

SP2/0 cells were cultured in RPMI-1640 medium (Weisen Biological Technology Co., Ltd., Nanjing, China) supplemented with 20% fetal bovine serum (FBS), 1% penicillin, and 100 µg/mL streptomycin at 37 °C and 5% CO_2_.

Vero cells were cultured under the same medium conditions but with 10% FBS, 1% penicillin, and 100 µg/mL streptomycin at 37 °C and 5% CO_2_.

### 2.2. Cloning, Expression and Purification of Recombinant PEDV N Protein

Based on published amino acid sequence of PEDV *N gene* from GenBank (CH-SWM-NC, PX482653.1, GIIa), target-specific primers for the *N gene* were designed using SnapGene software (v6.0.2; primers listed in Table 1). Total viral RNA was extracted from the intestinal tissue samples of diarrheic pigs using TRIzol reagent and subsequently reverse-transcribed into complementary DNA (cDNA). The *N gene* was then amplified from the cDNA template by PCR with PrimeSTAR^®^ DNA Max Polymerase (Takara, Tokyo, Japan), following the thermocycling conditions outlined in Table 2. The resulting PCR product was analyzed and purified via agarose gel electrophoresis, and then ligated into the pET-32a (+) vector that had been pre-digested with *BamH* I and *Not* I restriction enzymes. The constructed recombinant plasmid, designated pET-32a-N, was subsequently transformed into *E. coli* DH5α competent cells for propagation.

For protein expression, the recombinant plasmid was transformed into an expression host and induced with 1 mM isopropyl β-d-1-thiogalactopyranoside (IPTG) for 4 h. The expression and molecular identity of the recombinant protein were confirmed by sodium dodecyl sulfate-polyacrylamide gel electrophoresis (SDS-PAGE) and Western blotting analysis. Finally, the recombinant N protein was purified under native conditions using nickel-nitrilotriacetic acid (Ni-NTA) affinity chromatograph system, concentrated, and quantified using the bicinchoninic acid (BCA) assay.

### 2.3. Mice Immunization and Antiserum Preparation

The purified recombinant N protein was emulsified with an equal volume of Freund’s complete adjuvant. Female BALB/c mice (5–6 weeks old) were immunized via subcutaneous multipoint injection in the dorsal neck and back region, with each mouse receiving 100 µg N protein. For the subsequent second and third immunizations, the protein was emulsified with Freund’s incomplete adjuvant (FIA). Booster immunizations were administered at two-week intervals. One week after the final immunization, serum samples were collected from mice, and the titers of antigen-specific antibodies were determined by indirect ELISA. Mice exhibiting higher antibody titers were selected for a final booster immunization with 200 µg of the antigen (without adjuvant) to enhance the immune response.

### 2.4. Generation and Identification of Monoclonal Antibodies

Three days following the final immunization, splenocytes isolated from the immunized mice were fused with SP2/0 myeloma cells using polyethylene glycol (PEG) 4000 solution (P7181, Sigma, St. Louis, MO, USA). The fused cells were subsequently cultured in HAT selection medium. Ten days post-fusion, the culture supernatants were harvested and screened for specific antibodies by indirect ELISA to identify positive hybridoma clones. Stable monoclonal hybridoma lines were established through four rounds of limiting dilution subcloning. To produce antibodies in bulk, the positive hybridoma cells were intraperitoneally inoculated into BALB/c mice for the generation of ascitic fluid. The isotype of the resulting monoclonal antibodies (mAbs) was determined using a commercial mouse mAb isotyping kit (PK20003, Proteintech, Tokyo, Japan). The specificity of mAbs was further confirmed by Western blotting analysis and indirect immunofluorescence assay (IFA).

### 2.5. Indirect ELISA for Antibody Detection

The recombinant N protein or Carrier protein was diluted to 2 µg/mL in carbonate-bicarbonate buffer (0.05 M, pH 9.6) and was used to coat the wells of the ELISA plate by overnight incubation at 4 °C. Following coating, the plates were blocked with 5% (*w*/*v*) skimmed milk for 2 h at 37 °C. Subsequently, 100 µL of test samples, including hybridoma culture supernatants, mouse sera, and negative or positive control sera, were added to the wells and incubated for 1 h at 37 °C. Then, the plates were incubated with horseradish peroxidase (HRP)-conjugated goat anti-mouse IgG (diluted 1:5000) for 45 min at 37 °C. The colorimetric reaction was developed by adding 100 µL TMB substrate solution per well and incubating for 15 min at 37 °C in the dark. The reaction was terminated with 50 µL stop solution (2 M H_2_SO_4_), and the optical density was immediately measured at 450 nm (OD_450_) using a microplate reader (Ex1800, BioTek, Winooski, VT, USA).

### 2.6. Preliminary Localization of Linear B-Cell Epitopes of PEDV N Protein

To identify B-cell epitopes recognized by N-protein-specific mAbs, the *N gene* was initially truncated into two fragments, namely N-1 and N-147. These fragments were amplified by PCR using primers containing *EcoR* V and *BamH* I restriction sites and subsequently cloned into the p3xFLAG-CMV-7.1 vector for eukaryotic expression. The reactivity of the expressed recombinant truncated N proteins with the specific mAbs was assessed by Western blotting, using an anti-FLAG tag antibody as a control.

For finer mapping, the progressively shorter *N gene* fragments (N–98 and N–49; followed by N–82 and N–65) were amplified with primers bearing *EcoR* I and *Sal* I sites and inserted into the prokaryotic expression vector pEGX-4T-1. The resulting GST-fusion proteins were expressed in a prokaryotic system and analyzed by Western blotting with the screened mAbs and an anti-GST tag antibody.

To definitively confirm the minimal epitope, two overlapping synthetic peptides (10- and 11-amino acids in length) were stepwise synthesized and conjugated to a bovine serum albumin (BSA) carrier Sangon Biotech Co., Ltd. (Shanghai, China). The antigenicity of these peptides was verified by Western blot analysis using the screened mAbs. All primers used for generating *N gene* truncations are listed in Table 1.

### 2.7. Western Blotting Anlysis

Protein samples were separated by SDS-PAGE using 12.5% or 15% gel, as required by the experiment, and subsequently electrophoretically transferred onto polyvinylidene difluoride (PVDF) membranes. The membranes were then blocked with 5% (*w*/*v*) skimmed milk for 2 h at room temperature. After blocking, the membranes were incubated with the primary mAbs overnight at 4 °C. Thereafter, the membranes were probed with an HRP-conjugated goat anti-mouse IgG antibody (BA1050, Boster Biological Technology, Wuhan, China) at 1:6000 dilution for 45 min at 37 °C. Finally, the immunoreactive bands were visualized using a chemiluminescence detection kit (170-5061, Bio-Rad, Hercules, CA, USA) and imaged with a Tanon 5200 Multi-imaging system (Tanon, Shanghai, China).

### 2.8. Immunofluorescence Assay (IFA)

An indirect IFA was performed to assess the reactivity of the monoclonal antibodies with PEDV. Vero cells were infected with PEDV at a multiplicity of infection (MOI) of 1, with uninfected cells serving as the negative control. Indirect immunofluorescence was performed using hybridoma cell culture supernatants. At 24 h post-infection, Vero cells were fixed with 4% paraformaldehyde for 10 min and subsequently permeabilized with cold methanol for an additional 10 min. After washing with PBS, the cells were incubated with hybridoma culture supernatant as the primary antibody at 4 °C for 12 h. This was followed by a 45 min incubation at 37 °C with a CoraLite594-conjugated goat anti-mouse IgG (H + L) secondary antibody (SA00013-3, Proteintech, Rosemont, Illinois, IL, United States). Nuclei were counterstained with DAPI. Finally, fluorescent images were captured using a Zeiss Axiovert A1 fluorescence microscope (Carl Zeiss AG, Oberkochen, Germany).

### 2.9. Bioinformatics Analysis

The structural and functional analysis of the identified B-cell epitopes was performed using a suite of bioinformatics tools. Sequence conservation for the epitopes of mAbs 1A3, 1A10, and 1G1 was analyzed using ESPript3.0 and web logo3 software. Additionally, the secondary structure of the corresponding epitopes was predicted using DNAstar Lasergene 11.1. A structural model of the PEDV N protein predicted by AlphaFold 3 was then employed to map the precise three-dimensional localization of these epitopes in PyMOL 2.6. The spatial features and potential biological functions of the localized epitopes were further examined.

## 3. Results

### 3.1. Expression and Purification of Recombinant PEDV N Protein

**Figure 1:**
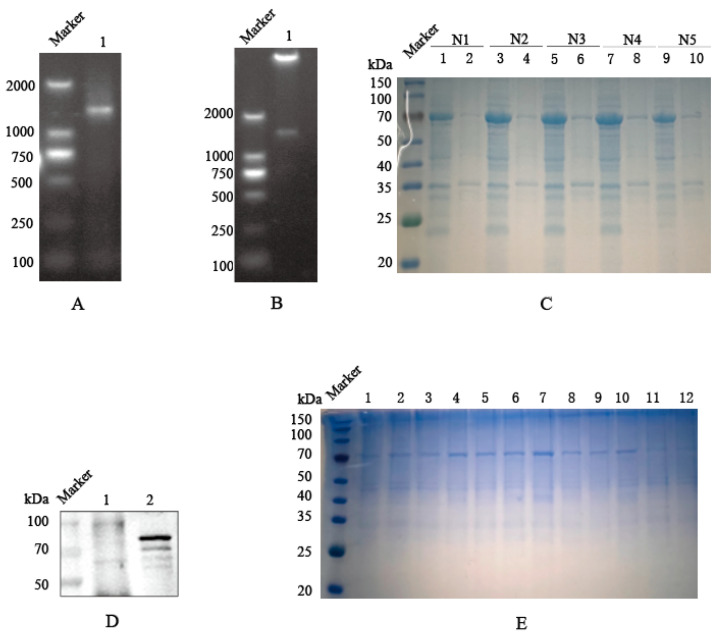
Prokaryotic expression of the N protein. (**A**) PCR amplification of *N gene*. Lane 1: Amplified *N gene* product; Lane Marker: 2 kb. (**B**) Identification of the recombinant plasmid pET-32a-N by double restriction enzyme digestion. Lane 1: Digested pET-32a-N fragment. (**C**) Expression analysis of N protein. The recombinant plasmid pET-32a-N was transformed into Rosetta cells, and protein expression was induced by IPTG, followed by SDS-PAGE analysis. Lanes 1, 3, 5, 7, and 9: Supernatants of five different pET-32a-N recombinant bacterial strains after ultrasonication; Lanes 2, 4, 6, 8, and 10: Insoluble pellets of the corresponding strains after ultrasonication. (**D**) Specific reactivity of the recombinant N protein with PEDV-positive serum. Lane 1: Negative control (pET-32a empty vector); Lane 2: Recombinant pET-32a-N (66.6 kDa). (**E**) Purification of the recombinant N protein. Lanes 1–12: Elution fractions collected at different imidazole concentrations. The imidazole concentrations were 100, 100, 100, 120, 120, 120, 150, 150, 150, 250, 250, and 250 mM.

The full-length PEDV *N gene* was successfully amplified by PCR, and agarose gel electrophoresis confirmed the amplification matched the expected size of 1326 bp (Figure 1A). Double enzyme digestion of the recombinant pET-32a-N plasmid verified the correct insertion of the *N gene* fragment (Figure 1B). SDS-PAGE analysis revealed that the recombinant protein had an approximate molecular weight of 66.6 kDa (Including the Trx fusion tag) and was predominantly expressed in the soluble fraction (Figure 1C). The antigenic identity was confirmed by Western blot analysis using PEDV-positive swine serum, which detected a specific band at the expected size of 66.6 kDa (Figure 1D). The recombinant N protein was subsequently purified via nickel-affinity chromatography. SDS-PAGE analysis of the elution fraction, collected at an imidazole concentration of 100–150 mM, showed a single prominent band corresponding to the target protein (Figure 1E). The final protein concentration was determined to be 0.399 mg/mL using the BCA assay.

### 3.2. Generation and Characterization of Anti-N Protein mAbs

**Figure 2:**
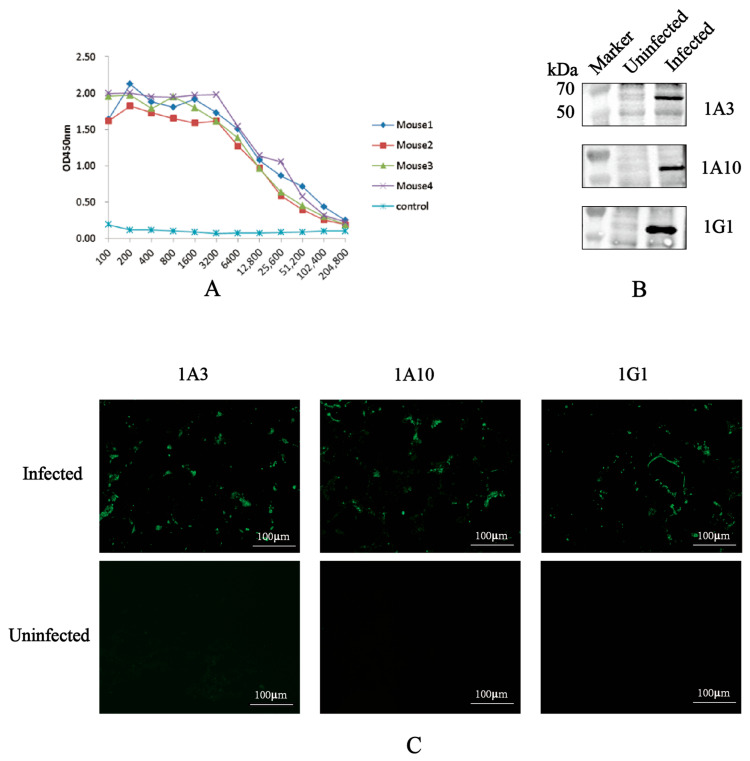
Screening and identification of monoclonal antibodies. (**A**) Antibody levels in immunized mice. Serum antibody titers from five immunized mice were measured by indirect ELISA one week after the third immunization. Results are presented as OD_450_ values. (**B**) Recognition of N protein from PEDV-infected cells by mAbs 1A3, 1A10, and 1G1. Vero cells were infected with PEDV, and protein samples were collected at 24 h post-infection. The ability of mAbs to recognize N protein was assessed by Western blot analysis. (**C**) Reactivity of three mAbs detected by IFA. Vero cells infected with PEDV were analyzed using IFA to determine the reactivity of the mAbs with N protein. The N protein was marked in green.

The serum antibody levels of the immunized mice were measured by indirect ELISA, as shown in Figure 2A. Mouse #4, which exhibited the highest antibody titer, was selected for a final booster immunization and subsequently used for hybridoma generation. Following cell fusion and four rounds of limiting-dilution subcloning, three hybridoma cell lines—designated 1A3, 1A10, and 1G1—were selected based on their stable secretion of N protein-specific antibodies, as determined by indirect ELISA using both the recombinant protein and the carrier protein as coating antigens. Isotype analysis classified all three mAbs as IgG1 isotype with kappa light chains (Table 3).

The specificity of these screened mAbs for N protein was confirmed by Western blot analysis. When probed with the hybridoma culture supernatants, lysates from PEDV-infected Vero cells showed a distinct band at approximately 55 kDa, corresponding to the viral N protein (Figure 2B). Furthermore, IFA results demonstrated strong reactivity of all three mAbs with PEDV-infected cells (Figure 2C), confirming their capability to recognize the native N protein in a cellular context.

### 3.3. Fine Mapping of Linear B-Cell Epitopes on PEDV N Protein

**Figure 3:**
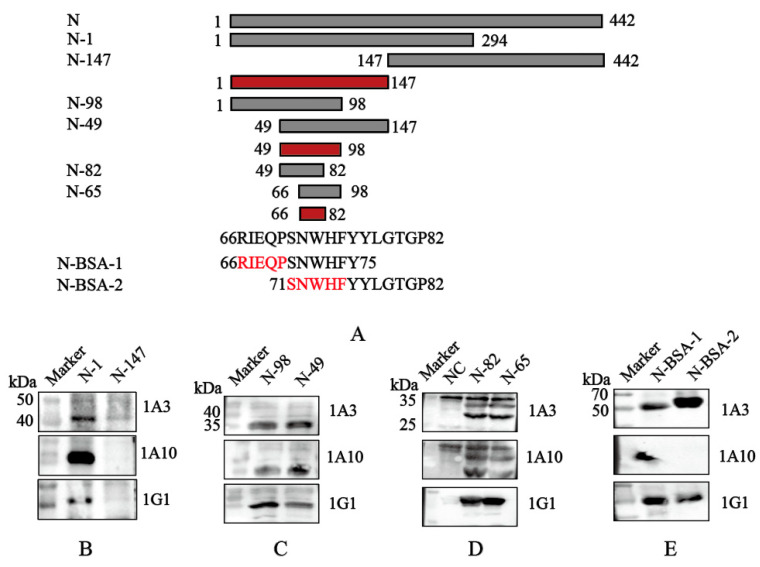
Identification of B-cell epitopes recognized by mAbs 1A3, 1A10, and 1G1. (**A**) Schematic diagram of N protein epitope mapping. The red highlighted regions indicate the final identified antigenic epitope fragments. (**B**) Recognition of truncated proteins N-1 and N-147 by mAbs 1A3, 1A10, and 1G1. (**C**) Recognition of truncated proteins N-98 and N-49 by mAbs 1A3, 1A10, and 1G1. (**D**) Recognition of truncated proteins N-82 and N-65 by mAbs 1A3, 1A10, and 1G1. (**E**) Recognition of the conjugated peptides by mAbs 1A3, 1A10, and 1G1.

To map the epitopes recognized by screened mAbs 1A3, 1A10, and 1G1, a series of six truncated N protein fragments and two synthetic peptides were progressively constructed (Figure 3A). Initially, the *N gene* was segmented into two truncated fragments: N-1 (amino acids 1–294) and N-147 (amino acids 147–442). These fragments were cloned into the p3xFLAG-CMV-7.1 vector for eukaryotic expression, and Western blot analysis showed that all three mAbs recognized N-1 protein (Figure 3B). For finer mapping, two shorter fragments, N-98 (amino acids 1–98) and N-49 (amino acids 49–147), were cloned into the prokaryotic expression vector pEGX-4T-1. Both recombinant proteins were recognized by the three mAbs (Figure 3C). Subsequently, even shorter fragments N-82 (amino acids 49–82) and N-65 (amino acids 66–98) were constructed and expressed as amino acids. Western blot confirmed that all mAbs recognized both N-82 and N-65 fragments (Figure 3D).

To determine the minimal epitope, two overlapping peptides ^66^RIEQPSNWHF^75^ and ^71^SNWHFYYLGTG^82^ were synthesized and conjugated to BSA. Western blot analysis demonstrated that peptide ^66^RIEQPSNWHF^75^ was recognized by mAbs 1A3, 1G1, and 1A10, while peptide ^71^SNWHFYYLGTG^82^ was recognized by mAbs 1A3 and 1G1 (Figure 3E). These results indicate that the core epitope for mAbs 1A3 and 1G1 might be centered on residues ^71^SNWHF^75^, whereas mAb 1A10 primarily recognizes residues ^66^RIEQP^70^.

### 3.4. Conservation Analysis of the Identified B-Cell Epitopes

**Figure 4:**
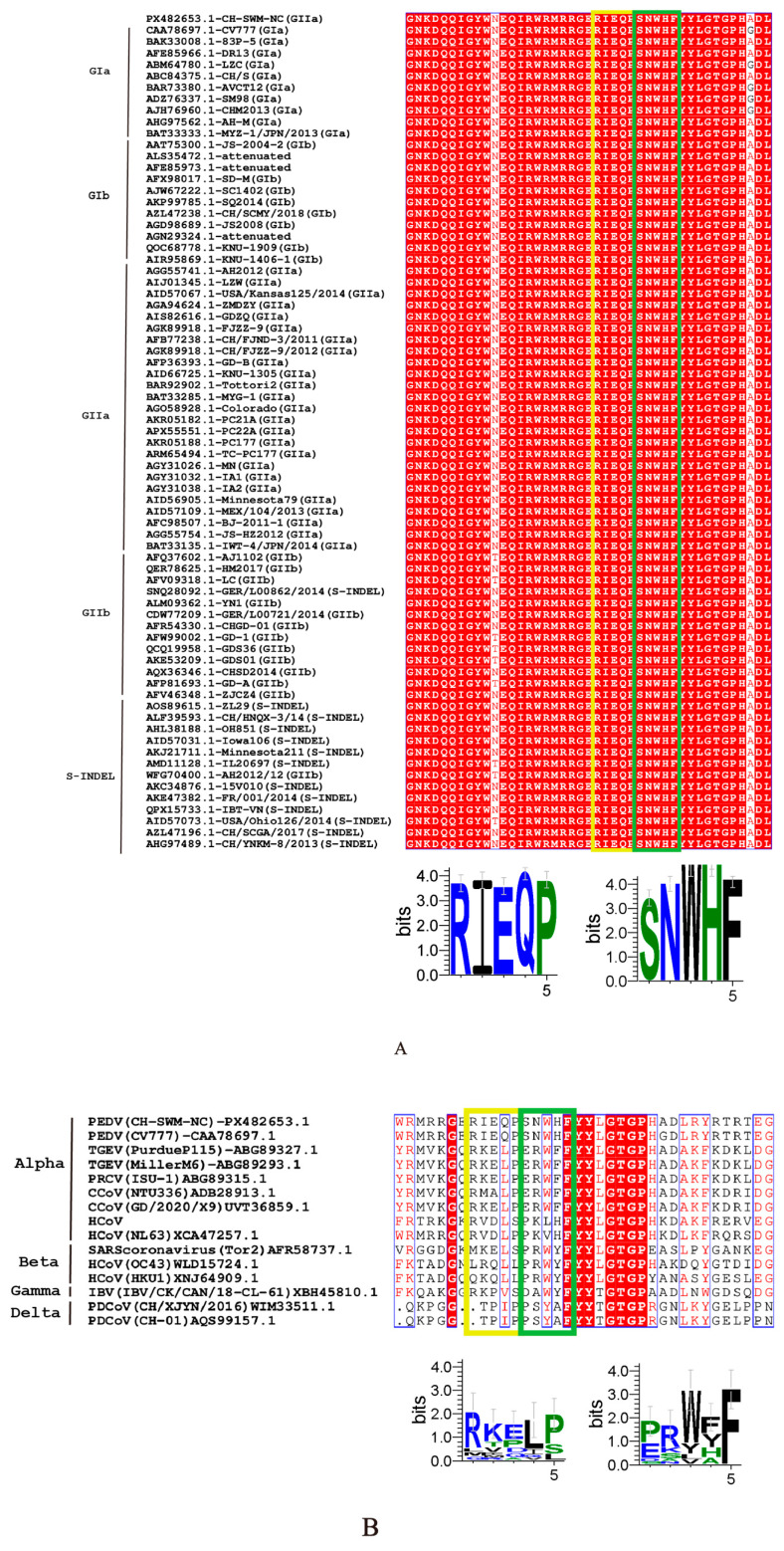
Comparison of antigenic epitopes on the N protein. (**A**) Homology analysis of 73 viral strains was performed using ESPript 3.0 and WebLogo 3.0. (**B**) Homology analysis of sequences from different coronaviruses was conducted using ESPript 3.0 and WebLogo 3.0. The antigenic epitope ^66^RIEQP^70^ is highlighted with a yellow box, and the epitope ^71^SNWHF^75^ is highlighted with a green box.

To evaluate the conservation of the epitopes recognized by mAbs 1A3 and 1G1, homology analyses were performed across different PEDV subtypes and among various coronaviruses. As shown in Figure 4A, both epitopes ^66^RIEQP^70^ and ^71^SNWHF^75^ were present in the N proteins of all 72 PEDV strains analyzed, indicating that these epitopes are highly conserved within PEDV. In contrast, alignment of the corresponding protein sequences from different coronaviruses revealed substantial divergence in these two epitopic regions (Figure 4B).

### 3.5. Spatial Localization Prediction of the Identified Epitopes

**Figure 5:**
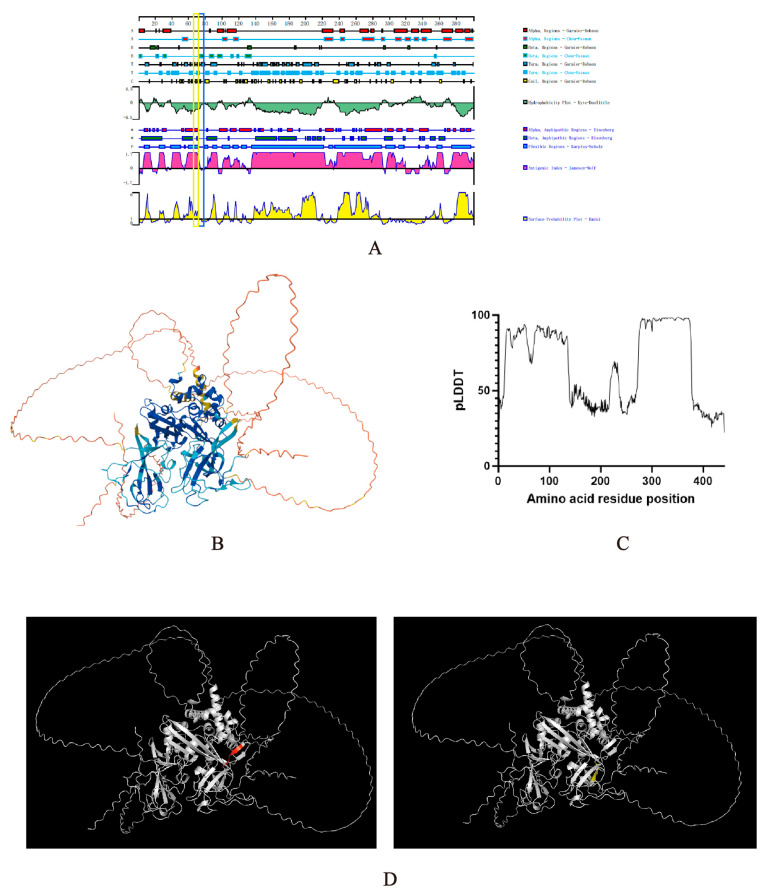
Localization analysis of the identified N-protein-specific epitopes. (**A**) Prediction of B-cell epitopes as antigenic targets using DNAstar Lasergene 11.1 software. The antigenic epitope ^66^RIEQP^70^ is highlighted with a yellow box, and the epitope ^71^SNWHF^75^ is highlighted with a blue box. (**B**) Three-dimensional structure of the PEDV N protein predicted by AlphaFold3, with residues color-coded according to pLDDT confidence scores. (**C**) line graph of per-residue pLDDT scores predicted by AlphaFold3. (**D**) Spatial localization of epitopes ^66^RIEQP^70^ and ^71^SNWHF^75^ within the PEDV N protein, visualized using PyMOL software and highlighted in red and yellow, respectively.

Based on predictions using DNAstar Lasergene 11.1 software (Figure 5A), the PEDV N protein epitope ^71^SNWHF^75^ is predominantly located within a regular structure comprising β-sheets. Both ^66^RIEQP^70^ and ^71^SNWHF^75^ are situated in regions with high antigenic index and hydrophilicity. The spatial localization of the two epitopes, ^66^RIEQP^70^ and ^71^SNWHF^75^, was analyzed using PyMOL2.6 software based on the three-dimensional structure of the N protein predicted by AlphaFold 3 (Figure 5B). The ^66^RIEQP^70^ epitope (90 > pLDDT > 70) is predicted to be exposed on the protein surface, whereas the ^71^SNWHF^75^ epitope (90 > pLDDT > 70) is predicted to be partially buried—findings that are consistent with the predictions generated by DNAstar.

## 4. Discussion

In recent years, PED has emerged as a severe threat to the swine industry. While early outbreaks were primarily associated with classical strains, contemporary circulating strains frequently exhibit genetic variations. Conventional vaccines based on the classical strain CV777 have shown limited efficacy in protecting pigs in China, underscoring the urgent need for accurate early diagnostic techniques and novel vaccines for PEDV prevention [20,21,22].

The PEDV S protein is a surface-exposed glycoprotein that induces neutralizing antibodies critical for virus neutralization [23]. In contrast, the N protein is located inside the virion, and antibodies targeting N protein lack neutralizing activity [24]. However, the S protein exhibits high genetic variability among different PEDV strains, which limits its suitability as a broad-spectrum diagnostic target. The PEDV N protein, being highly abundant, immunogenic, and conserved during viral infection, serves as an ideal target for diagnostic development, highlighting the importance of characterizing N protein-specific mAbs and their epitopes, and represents a promising target for such applications [25].

Here, we prokaryotically expressed and purified the PEDV N protein, and used it to generate three monoclonal antibodies (1A3, 1A10, and 1G1) from immunized mice. These mAbs were confirmed to specifically bind the native N protein via Western blot and IFA. A detailed epitope mapping strategy employing sequential truncations and synthetic peptides identified two novel linear B-cell epitopes: ^66^RIEQP^70^ (recognized by mAb 1A10) and ^71^SNWHF^75^ (recognized by mAbs 1A3 and 1G1).

These epitopes are highly conserved across diverse PEDV strains, including the prevalent GII genotype, but differ markedly in the corresponding homologous regions of other coronaviruses. From an application perspective, such highly specific monoclonal antibodies hold significant value for the clinical diagnosis of PEDV.

To further contextualize the newly identified epitopes within the established antigenic landscape of the PEDV N protein, we compared the distribution of epitopes ^66^RIEQP^70^ and ^71^SNWHF^75^ with those previously reported (Table 4). The analysis revealed that epitope ^66^RIEQP^70^ is located within the N-terminal domain (NTD) of the N protein—a region known to contain multiple linear epitopes, including ^12^KRVPLSLY^19^ [26] and ^56^QIRWRMRRGERI^67^ [27]. Although overlapping peptides encompassing ^66^RIEQP^70^ have been mentioned in earlier studies, epitope ^71^SNWHF^75^, as an independent functional linear epitope, has not yet been systematically identified or functionally validated. Therefore, ^71^SNWHF^75^ represents a previously unreported novel linear epitope.

In this study, structural modeling revealed that epitope ^66^RIEQP^70^ is exposed on the protein surface, while ^71^SNWHF^75^, although partially buried, retains favorable solvent accessibility. Notably, the three monoclonal antibodies obtained target two adjacent linear epitopes within the 66–75 region of the N protein. This region exhibits high hydrophilicity and a high antigenic index, which may represent an immunodominant feature during the murine immune response, leading to its targeting by multiple independent B-cell clones.

In future studies, we will further investigate the specificity of these three monoclonal antibodies based on the comparative sequence alignment of coronaviruses. We aim to confirm their high specificity for PEDV and eliminate potential cross-reactivity with TGEV and other related viruses, which often cause diagnostic confusion in clinical settings. Ultimately, these well-characterized mAbs will serve as bioreagents for the development of diagnostic methods exclusively specific to PEDV.

In summary, this study provides three well-characterized mAbs and defines two novel, conserved linear B-cell epitopes using a stepwise truncation strategy. These findings provide valuable tools and insights for developing epitope-based PEDV diagnostics and further investigating the functional role of N protein.

## Figures and Tables

**Table 1:** Primers of the truncated *N gene* for PCR cloning.

Gene Name	Primer Name	Primer Sequence (5′–3′)	Primer Size	Product Size
N	N-F	ATCGGATCCATGGCTTCTGTCAGCTTTCAGGA	32	1346 bp
	N-R	AGTGCGGCCGCTTAATTTCCTGTATCGAAGATCTCGT	37	
N-1	N-1-F	CTGATATCAATGGCTTCTGTCAGCTTTCAG	30	605 bp
	N-1-R	CGGATCCGTTATTATTATTGCCTCCTCTGTTCTG	35	
N-147	N-147-F	CTGATATCAGCTAAAGAAGGCGCAAAGACT	30	605 bp
	N-147-R	CGGGATCCGCAAGCTGCTACGCTATTTTC	29	
N-98	N-98-F	CGGAATTCATGGCTTCTGTCAGCTTTCAG	29	310 bp
	N-98-R	GAGTCGACAACCCAGAAAACACCCTCAGT	29	
N-49	N-49-F	CCCGGAATTCATTGGATACTGGAATGAGCAA	31	312 bp
	N-49-R	GAGTCGACCGAATTTGCACGTGAAGTAGG	29	
N-82	N-82-F	CGGAATTCATTGGATACTGGAATGAGCAA	29	112 bp
	N-82-R	GAGTCGACTCCTGTTCCGAGGTAGTAGAA	29	
N-65	N-65-F	CGGAATTCCGAATTGAACAACCTTCCAAT	29	115 bp
	N-65-R	GAGTCGACAACCCAGAAAACACCCTCAGT	29	

Note: Horizontal characters are restriction endonuclease sites, as follows: GGATCC, *BamH* I; GCGGCCGC, *Not I*; GATATC, *EcoR* V; GAATTC, *EcoR* I; GTCGAC, *Sal* I.

**Table 2:** PCR reaction program.

Reaction Basic Steps	Temperature (°C)	Time	Cycles
Initial denaturation	98	10 min	1
Denaturation	98	10 s	35
Annealing	55	5 s	35
Extension	72	10 s	35
Final Extension	72	10 min	1

**Table 3:** Monoclonal antibody isotype identification and antibody potency.

mAbs	Antibody Potency of Ascites	Heavy Chain	Light Chain
1A3	1:204,800	IgG1	Kappa
1G1	1:204,800	IgG1	Kappa
1A10	1:102,400	IgG1	Kappa

**Table 4:** Reported Antigenic Epitopes of the PEDV Nucleocapsid Protein.

Epitope Position (Amino Acids)	Epitope Sequence	Type	Key Reference(s)
12–19	KRVPLSLY	Linear	Sun, M. et al., 2024 [28]
18–133	-	Conformational	Wang, K. et al., 2016 [29]
56–67	QIRWRMRRGERI	Linear	Wang, X. et al., 2020 [30]
133–140	EIVEPNTP	Linear	Zhao, Y. et al., 2023 [26]
155–162	GNNRSRSP	Linear	Zhao, Y. et al., 2023 [26]
223–235	VAAVKDALKSLGI	Linear	Yang, W. et al., 2019 [27]
235–242	IGENPDKL	Linear	Sun, M. et al., 2024 [28]
252–262	RSDSSGKNTPK	Linear	Wang, K. et al., 2016 [29]
274–282	DLKDIPEWR	Linear	Wang, J. et al., 2025 [31]
302–310	KNFGDAEFV	Linear	Zhao, Y. et al., 2023 [26]
318–342	GYAQIASLAPNVAALLFGGNVVRE	Linear	Wang X.et al., 2020 [30]
350–357	TYNYKMTV	Linear	Zhao, Y. et al., 2023 [26]
372–380	DAFKTGNA	Linear	Sun, M. et al., 2024 [26]
398–406	HEEAIYDDV	Linear	Wang, X. et al., 2020 [30]
402–409	IYDDVGVP	Linear	Zhao, Y. et al., 2023 [26]
413–420	THANLEWD	Linear	Zhao, Y. et al., 2023 [26]

## Data Availability

The data presented in this study are available upon request from the corresponding author.
